# Research on Optimizing Electronic Nose Sensor Arrays for Oyster Cold Chain Detection Based on Multi-Algorithm Collaborative Optimization

**DOI:** 10.3390/bios15120772

**Published:** 2025-11-25

**Authors:** Yirui Kong, Zhenhua Guo, Weifu Kong, Hongjuan Li, Xinrui Li, Xiaoshuan Zhang, Xinzhe Liu, Ruihan Wu, Baichuan Wang

**Affiliations:** 1 Yantai Institute, China Agricultural University, Yantai 264670, China; 2 Beijing Laboratory of Food Quality and Safety, College of Engineering, China Agricultural University, Beijing 100083, China; 3 Sanya Institute, China Agricultural University, Sanya 572025, China

**Keywords:** sensor array optimization, electronic nose, multi-algorithm collaborative optimization, oyster quality, cold chain transportation

## Abstract

Real-time quality monitoring during oyster cold chain transportation is a critical component in ensuring food safety. Addressing the issues of high redundancy and insufficient environmental adaptability in existing electronic nose systems, this study proposes a multi-algorithm collaborative optimization strategy for sensor array optimization. The system integrates ten gas sensors (TGS series, MQ series), employing Random Forest (RFA), Simulated Annealing (SA), and Genetic Quantum Particle Swarm Optimization (GA-QPSO) for sensor selection. KNN combined with K-means analysis validates the optimization outcomes. Under cold chain environments at 4 °C, 12 °C, 20 °C, and 28 °C, a multidimensional dataset was constructed by extracting global variables using feature correlation functions. Experiments demonstrate that the optimized sensor count decreases from 10 to 5–6 units while maintaining recognition accuracy above 95%, with redundancy decreased by over 40%. This multi-algorithm collaborative optimization effectively balances sensor array recognition precision, resource efficiency, and environmental adaptability, providing an intelligent, high-precision technical solution for oyster cold chain monitoring.

## 1. Introduction

Oysters, valued for their high nutritional and economic worth, have become a core commodity in the global seafood trade. Monitoring their quality during cold chain transportation is crucial for ensuring food safety and commercial value. However, traditional detection methods (such as manual sensory evaluation and high-performance liquid chromatography) suffer from limitations including low efficiency, high cost, and poor real-time capability. Particularly in cold chain logistics, the dynamic changes in volatile gases (amines, alkanes, etc.) released during oyster spoilage make it difficult for traditional methods to achieve rapid, non-destructive real-time detection, thereby increasing the risk of quality deterioration [1,2,3,4].

Electronic nose technology employs gas sensor arrays and pattern recognition algorithms to intelligently identify volatile gases [5,6,7,8,9]. However, existing systems commonly suffer from high sensor redundancy. Redundant sensors not only introduce noise and increase computational complexity and costs but also hinder practical application. Previous studies have employed single optimization algorithms (e.g., genetic algorithms) to streamline sensor arrays, but these strategies are limited to static environments or single objectives [10]. Moreover, single algorithms are prone to local optima or insufficient convergence, struggling to adapt to the dynamic effects of temperature fluctuations on gas components in cold chain scenarios [6,11].

To address these limitations, this study proposes a multi-algorithm collaborative optimization framework. It integrates Random Forest (RFA), Simulated Annealing (SA), and Genetic Quantum Particle Swarm Optimization (GA-QPSO) with a clustering validation strategy. This approach enables dynamic sensor array reduction and performance enhancement while balancing global search capability, environmental adaptability, and computational efficiency, providing a solution for efficient detection in multi-temperature zones [7]. Beyond addressing these technical challenges, optimizing electronic-nose systems for oyster cold-chain monitoring has significant industrial and scientific implications. It enables improved traceability and safety assurance in aquatic-product logistics, supports real-time quality decision-making during transportation, and contributes to developing intelligent food-safety management frameworks for perishable goods.

## 2. Materials and Methods

### 2.1. Experimental Materials

Freshly harvested oysters were purchased from an oyster farming base in Yantai, China. The oysters were uniformly sized and available in sufficient quantities. After removing half the shells, the oysters were stored in ice-filled foam boxes and promptly transported to the laboratory. After shell removal, the oysters were rinsed with sterile saline to remove surface mucus. They were placed in modified polypropylene preservation boxes (19 cm × 12.5 cm × 7.5 cm) equipped with humidity controllers (maintaining 85% RH) to prevent sample dehydration and ensure optimal volatile gas release.

### 2.2. Electronic Nose System

Single-gas sensors struggle to meet the complex detection demands of multi-component gases in oyster cold chains due to insufficient selectivity and sensitivity [12,13,14]. This study employs a cross-sensitive sensor array combined with pattern recognition technology, leveraging the differentiated response characteristics of multiple sensors (TGS and MQ series) to achieve efficient identification and quantitative analysis of gas components. Based on target gas characteristics, cost, and integration compatibility, an optimized array comprising 10 sensors (Figaro TGS series from Japan and Winsen MQ series from China) was constructed (Table 1). Its multi-source information fusion capability significantly enhances the detection system’s specificity and environmental adaptability.

### 2.3. Test Methods

#### Electronic Nose Detection Test Protocol

**Figure 1:**
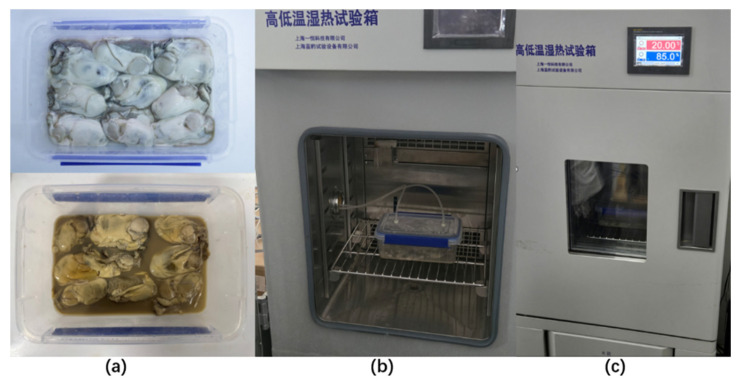
Experimental setup for electronic nose detection. (**a**) Fresh oysters used as test samples. (**b**) Placement of oysters inside the temperature–humidity chamber under controlled cold-chain simulation. (**c**) Data acquisition process showing airflow pathway and sensor array connection. All tests were conducted at four temperature conditions (4 °C, 12 °C, 20 °C, 28 °C) with 85% RH to simulate cold-chain storage.

This test design establishes a standardized data acquisition process to meet the dynamic quality monitoring requirements for oyster cold chains. Each test cycle comprises a 20-min gas pathway cleaning phase followed by five repeated sampling sessions (5 min per sample at 1 Hz frequency). The gas pathway selection module controls the switching between sample and cleaning gas streams to ensure unobstructed airflow and prevent cross-contamination. Experimental setup for electronic nose detection is shown in Figure 1. Four temperature conditions (4 °C, 12 °C, 20 °C, 28 °C) were set, with each condition undergoing three independent replicate tests to validate result reliability. The four temperature conditions (4 °C, 12 °C, 20 °C, 28 °C) were selected to represent typical oyster cold-chain logistics stages and spoilage thresholds reported in previous studies. Specifically, 4 °C corresponds to the refrigeration stage used during transport and retail storage; 12 °C represents mild temperature fluctuation during unloading and handling; 20 °C approximates room-temperature exposure, and 28 °C reflects accelerated spoilage or microbial growth conditions commonly used for shelf-life simulation. These temperature points have been widely adopted in oyster quality and microbial activity studies. The specific procedure is as follows:

(1) Sensor Pre-calibration: After array integration into the system, continuous preheating for 7 days ensures response stability.
(2) Sample Preparation and Environmental Simulation: Place 9 oysters in a sealed polypropylene chamber (19 cm × 12.5 cm × 7.5 cm) and simulate cold chain temperature gradients using a temperature and humidity test chamber.
(3) Data Acquisition and Control: The chamber interfaces with the detection system to initiate closed-loop airflow control. The host computer triggers continuous data collection until sample spoilage occurs.
(4) Data Processing: Data from three replicate experiments are categorized, labeled, and averaged. The dynamic changes in volatile gas components are analyzed for their correlation with product quality.

The temperature–humidity chamber used in this study (LHS-150CL, Shanghai Bluepard Instruments Co., Ltd., Shanghai, China) was designed to replicate the dynamic conditions of oyster cold-chain storage. During the experiment, temperature and relative humidity were continuously monitored and automatically controlled (±0.5 °C and ±3% RH, respectively) through an internal circulation system equipped with air inlets and exhaust vents to maintain uniform airflow and prevent gas accumulation. The modified polypropylene preservation boxes were not completely airtight; each box contained a microporous vent (2 mm diameter) to allow for limited gas exchange, simulating oxygen permeability and CO_2_ release during refrigerated transport. The airflow rate inside the chamber was maintained at 0.3–0.5 m s^−1^, comparable to the gentle air circulation in refrigerated logistics vehicles. This configuration ensured that temperature and humidity fluctuations closely approximated those observed in practical cold-chain storage and transportation conditions.

To ensure consistency and reproducibility of the collected signals, all raw sensor response data were preprocessed before feature extraction. A mean-averaging operation was first applied to each sampling period to minimize short-term fluctuations. Signal noise was then smoothed using a Savitzky–Golay filter with a window size of 11 and a third-order polynomial, which effectively reduced high-frequency noise while preserving the overall response curve shape. Subsequently, data were normalized using the Z-score method, defined as

(1) $$x^{\prime }=\frac{x-\mu }{\sigma }$$

where *x* represents the raw response value, and *μ* and *σ* denote the mean and standard deviation of each sensor signal under the same temperature condition. Independent normalization parameters were computed for each temperature condition (4 °C, 12 °C, 20 °C, and 28 °C) to maintain temperature-specific signal comparability. Outliers were identified using the three-sigma (3σ) criterion, and samples containing more than 5% abnormal points within a measurement cycle were excluded from further analysis. This standardized preprocessing procedure ensured that the subsequent feature extraction and optimization processes were based on clean, stable, and comparable datasets across different temperature environments.

### 2.4. Optimization of Electronic Nose Sensor Array

#### 2.4.1. Sensor Array Optimization Method

**Figure 2:**
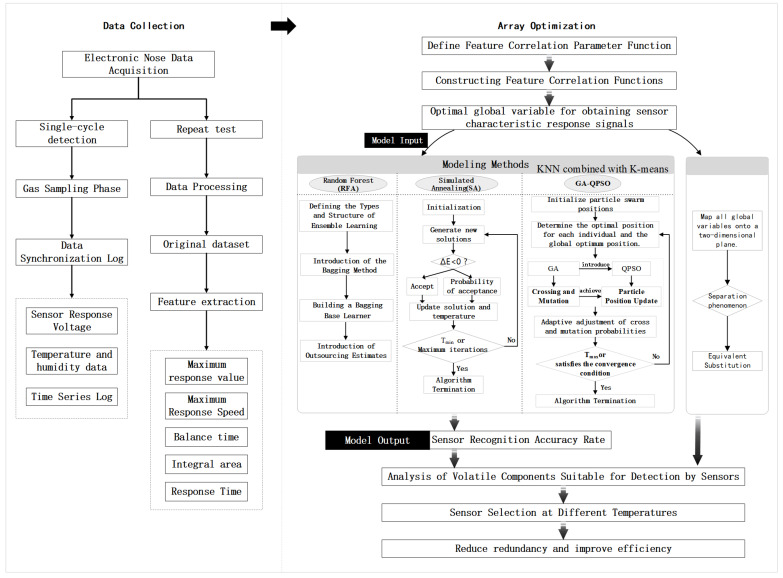
Sensor Array Optimization Scheme.

This study employs the following systematic approach to achieve optimized sensor array design: First, establish a feature correlation function based on defined feature correlation parameters to obtain the optimal global variables for the feature response signals of 10 sensors (S1–S10). Second, multidimensional data modeling was achieved through a dual-path analysis framework: Path One employed the optimal global variables of feature response signals as machine learning input parameters, utilizing RFA, SA, and GA-QPSO algorithms for model training and validation, with sensor recognition accuracy as the output metric. Path Two employs KNN and K-means clustering algorithms to reveal sensor feature clustering effects through two-dimensional feature space mapping, establishing equivalent classification criteria for alternative sensors [15]. Based on this foundation, the system evaluates the detection efficacy and specificity of each sensor for different compound categories. By integrating temperature gradient tests (4, 12, 20, 28 °C) and conducting a multidimensional performance indicator fusion analysis, it determines the optimal sensor combination scheme for each temperature range. This achieves synergistic optimization of the sensor array across three dimensions: identification accuracy, resource utilization efficiency, and environmental adaptability. The specific scheme is illustrated in Figure 2.

#### 2.4.2. Construction of Feature Correlation Functions

**Figure 3:**
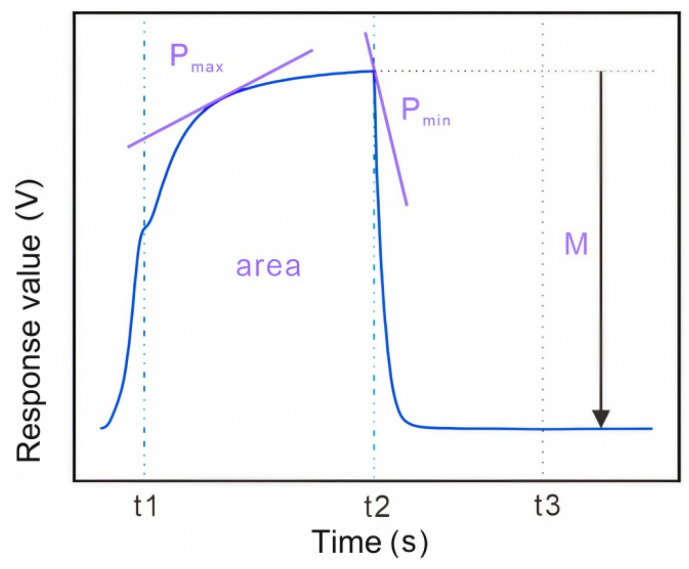
Definition of Feature Correlation Parameter Function.

This study proposes parameter function support for feature correlation extraction. The specific definitions of parameter functions are shown in Figure 3:

(a) Maximum response value *Pmax*:

(2) $$\mathrm{Pmax}=\frac{\mathrm{dp}\left(t\right)}{\mathrm{dt}}\max$$

(b) Maximum response value *Pmin*:

(3) $$\mathrm{Pmin}=\frac{\mathrm{dp}\left(t\right)}{\mathrm{dt}}\min$$

(c) Reaction equilibrium time *T*
(d) Response surface integral *area*:

(4) $$\mathrm{area}=\frac{d^{2}S\left(t\right)}{dt^{2}}$$

(e) Response time: *t*1, *t*2, *t*3

The definition of the feature correlation function uses the response characteristic curves of the previous 10 sensor signals as the baseline. Here, (a) to (e) are defined as global variables for feature optimization.

The feature correlation function is used to quantify the similarity or correlation between the response characteristic curve of a sensor signal and the target feature. The calculation formula for the feature correlation function is as follows:

(5) $$C\left(a,b,c,d,e\right)=\sum_{i=1}^{10}\;w_{i}\cdot \mathrm{Similarity}\left(F_{i},F_{\mathrm{target}}\left(a,b,c,d,e\right)\right)$$

where $F_{i}$ denotes the response characteristic curve of the i-th sensor signal; $F_{\mathrm{target}}(a,b,c,d,e)$ denotes the target characteristic curve generated based on global variables (a,b,c,d,e); $w_{i}$ denotes the weight of the i-th sensor signal, used to adjust the importance of different sensors; Similarity (.) denotes the similarity metric function, which can be Euclidean distance, cosine similarity, correlation coefficient, etc.

The target feature curve $F_{\mathrm{target}}(a,b,c,d,e)$ can be generated using the following formula:

(6) $$F_{\mathrm{target}}\left(t;a,b,c,d,e\right)=a\cdot f\left(c\cdot \left(t-b\right)\right)+e\cdot \mathrm{Noise}\left(t\right)$$

where t denotes the time variable, f(·) represents the functional form of the reference characteristic curve, and Noise(t) denotes the noise function used to simulate random noise in sensor signals.

The objective of feature optimization is to find a set of global variables (a,b,c,d,e) that maximizes the feature correlation function C(a, b, c, d, e). That is: maximize the number C(a,b,c,d,e). That is:

(7) $$\left(a*,b*,c*,d*,e*\right)=\mathrm{ar}g\underset{a,b,c,d,e}{\max}\;C\left(a,b,c,d,e\right)$$

By defining feature correlation functions and global variables (a,b,c,d,e), the similarity between the sensor signal response characteristic curve and the target features can be quantified. Then the optimal global variables can be identified by an optimization method.

#### 2.4.3. Sensor Array Optimization Modeling Method Based on Feature Correlation Functions

**(1)**  **Random Forest (RFA):** Based on ensemble learning theory, multiple independent decision tree models are generated via the Bagging method. The base learners output sensor recognition accuracy metrics, while out-of-bag estimates evaluate model generalization capabilities, ensuring robust optimization results [15,16,17,18].
**(2)**  **Simulated Annealing (SA):** Balances global exploration and local exploitation by incorporating a temperature decay function and probabilistic acceptance mechanism. Defines termination conditions to converge the algorithm toward the global optimum [19,20,21,22].
**(3)**  **GA-QPSO (Genetic Algorithm-Quantum Particle Swarm Optimization):** Integrates the global search capability and population diversity of genetic algorithms (GA) with the quantum behavior characteristics and rapid convergence advantages of quantum particle swarm optimization (QPSO). An adaptive update mechanism dynamically adjusts crossover and mutation probabilities to balance global exploration and local search capabilities [23,24].

The hybrid RFA–SA–GA-QPSO strategy was designed to combine the complementary advantages of ensemble learning, stochastic search, and intelligent swarm optimization. RFA (Random Forest Algorithm) provides robust noise resistance and feature importance estimation, serving as the baseline classifier to quantify sensor relevance under non-linear and noisy conditions. SA (Simulated Annealing) introduces probabilistic global search with a temperature decay mechanism, preventing premature convergence and enabling dynamic exploration of sensor combinations. GA-QPSO integrates the genetic algorithm’s population diversity with the quantum particle swarm’s rapid convergence and adaptive learning capability, enhancing fine-tuning efficiency during the late optimization stage.

The three algorithms operate in a sequential collaborative manner: RFA first evaluates and ranks sensors based on feature importance; SA then performs combinatorial optimization using these ranked features as the initial state; finally, GA-QPSO refines the search space and convergence toward the global optimum. This tri-level hybridization allows the model to achieve high robustness, global search ability, and convergence speed simultaneously, outperforming simpler dual-ensemble schemes such as RFA–PSO or GA–DE.

A schematic description of this algorithmic workflow has been added in the text to clarify the operational sequence and synergistic roles of each algorithm.

The three algorithms complement each other in noise resistance, global optimization, and dynamic adaptability: RFA provides a high-precision classification benchmark, SA addresses combinatorial search challenges, while GA-QPSO balances global optimization and rapid convergence through its hybrid strategy.

The classification accuracy (Acc, %) was computed as the ratio between the number of correctly classified samples (*N*_correct_) and the total number of samples (*N*_total_), i.e.,


$$\mathrm{Acc}(\%)=(N_{\mathrm{correct}}/N_{\mathrm{total}})\times 100$$

Each model’s accuracy was averaged across five independent runs under a 5 × 2 cross-validation protocol for each temperature condition (4 °C, 12 °C, 20 °C, 28 °C). Macro-accuracy was obtained as the mean of the accuracies across the four temperature zones.

The three-level hybrid optimization operates in a logically sequential and collaborative manner that reflects the functional complementarity of each algorithm. RFA first acts as a noise-resistant evaluator, ranking sensor features according to their importance and thereby providing an initial relevance landscape. The ranked information is then passed to SA, which performs stochastic global exploration based on probabilistic acceptance and temperature decay to identify high-potential sensor combinations and avoid local optima. Finally, GA-QPSO refines these candidate solutions through adaptive population evolution and quantum-inspired particle updates, enabling rapid convergence toward the global optimum. In this workflow, each algorithm contributes a distinct role—RFA ensures robustness and interpretability, SA enhances global search diversity, and GA-QPSO accelerates fine-grained convergence. Their sequential cooperation effectively unites robustness, exploration, and exploitation, leading to superior performance compared with simpler dual-algorithm schemes.

#### 2.4.4. KNN Combined with K-Means for Sensor Array Optimization

By integrating KNN with K-means, all optimal global variables are mapped onto a two-dimensional plane, revealing the clustering effect of sensor features and providing a theoretical basis for sensor equivalent replacement strategies [25,26,27].

#### 2.4.5. Analysis of Sensor Detection Performance for Target Gas Components

Based on the identification results of key components in oyster cold chain quality (alcohols, aldehydes, alkanes, etc.), we can analyze the detection performance of sensors for different target gas components (specific response capability, detection range) as well as the similarity and complementarity among sensors.

## 3. Results and Analysis

### 3.1. Sensor Array Optimization Results

#### 3.1.1. Sensor Array Optimization Results for Three Models

**Figure 4:**
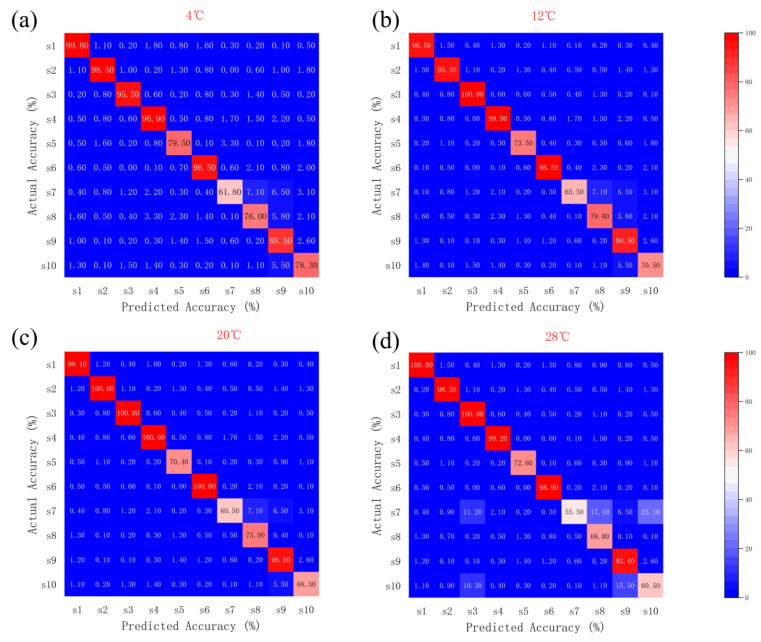
Recognition accuracy of feature importance identification for the ten sensors using the Random Forest Algorithm (RFA) at four temperature conditions. (**a**) 4 °C, (**b**) 12 °C, (**c**) 20 °C, (**d**) 28 °C. The *x*-axis represents individual sensors (S1–S10), and the *y*-axis indicates classification accuracy (%).

As shown in Figure 4, the Random Forest Algorithm (RFA) exhibited high recognition accuracy across all temperature zones, generally above 95%, with S3 and S4 showing the most stable performance (≥95% from 4 °C to 28 °C). Minor variations were observed among sensors at elevated temperatures, but overall accuracy remained consistently high.

**Figure 5:**
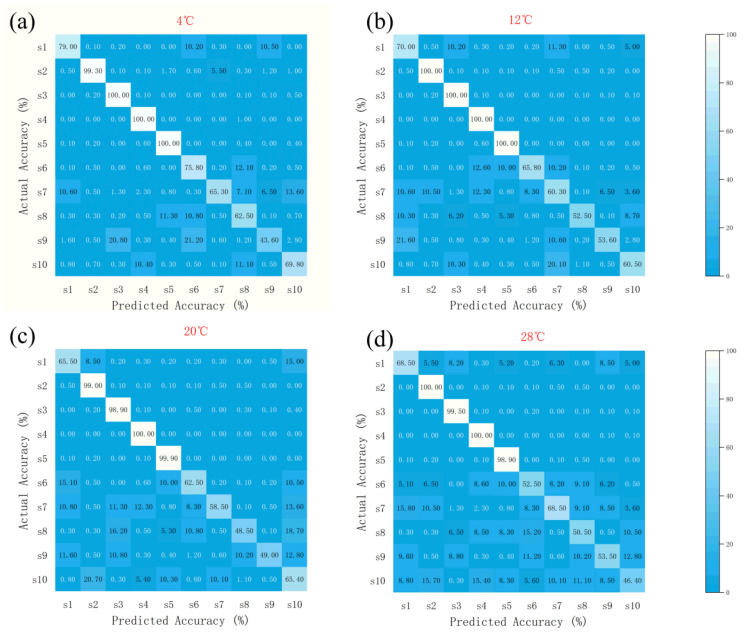
Accuracy of Feature Importance Identification for 10 Sensors at Different Temperatures (SA). (**a**) 4 °C, (**b**) 12 °C, (**c**) 20 °C, (**d**) 28 °C. The *x*-axis represents individual sensors (S1–S10), and the *y*-axis indicates classification accuracy (%).

SA Optimization Results (Figure 5): At 4 °C, the recognition accuracy rates for S2, S3, S4, and S5 were 99.3%, 100%, 100%, and 100%, respectively; all reached 100% at 12 °C; at 20 °C, they were 99%, 98.9%, 100%, and 99.9%; and at 28 °C, they were 100%, 99.5%, 100%, and 98.9%, respectively. S3 and S4 demonstrated outstanding stability across the entire temperature range (accuracy ≥ 98.9%), while S5 showed only a slight decrease to 98.9% at 28 °C.

**Figure 6:**
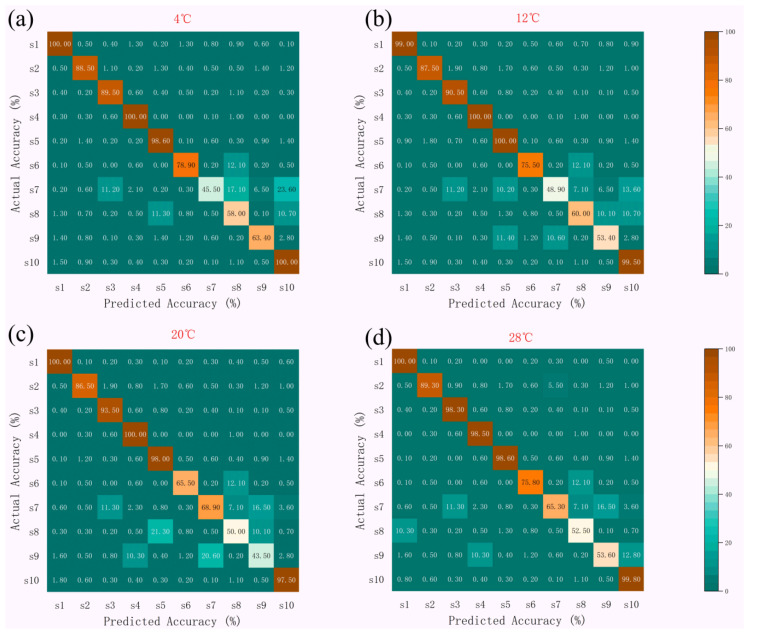
Accuracy of Feature Importance Identification for 10 Sensors at Different Temperatures (GA-QPSO). (**a**) 4 °C, (**b**) 12 °C, (**c**) 20 °C, (**d**) 28 °C. The *x*-axis represents individual sensors (S1–S10), and the *y*-axis indicates classification accuracy (%).

GA-QPSO Results (Figure 6): At 4 °C, the recognition accuracies of S1, S4, S5, and S10 were 100%, 100%, 98.6%, and 100%, respectively; At 12 °C, the accuracy rates for S1, S3, S4, S5, and S10 were 99%, 90.5%, 100%, 100%, and 99.5%, respectively; at 20 °C, S3, S5, and S10 showed slight fluctuations (93.5%, 98%, 97.5%), while S1 and S4 maintained 100%; At 28 °C, the precision rates for S1, S3, S4, S5, and S10 were 100%, 98.3%, 98.5%, 98.6%, and 99.8%, respectively. S1 and S4 demonstrated outstanding performance across the entire temperature range (accuracy ≥ 98.5%). S3 exhibited the lowest performance at 12 °C (90.5%) but recovered significantly as temperature increased.

Based on the optimization results of the above three algorithm models, the sensor types with good recognition performance at the four temperatures are shown in Table 2.

#### 3.1.2. Optimization Results of Sensor Arrays Using KNN Combined with K-Means Algorithm

**Figure 7:**
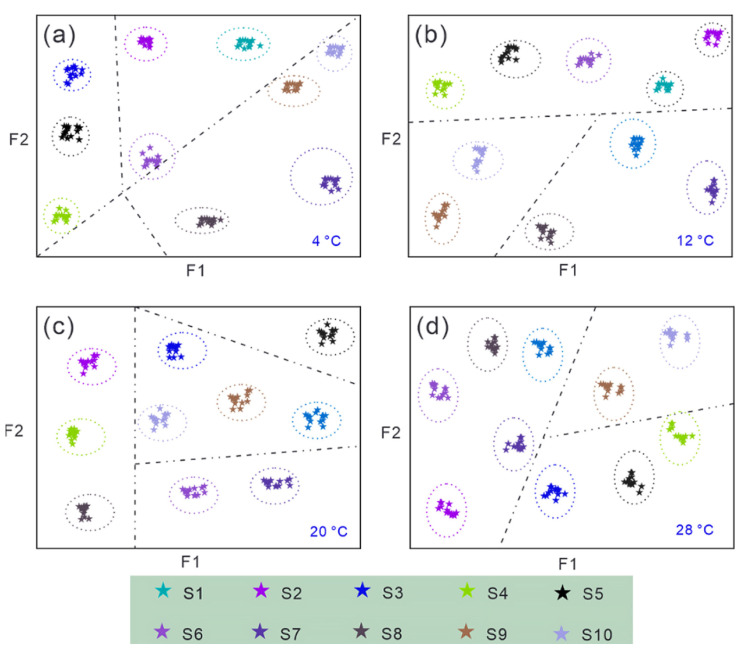
Optimized global variables for 10 sensors at different temperatures. (**a**) 4 °C, (**b**) 12 °C, (**c**) 20 °C, (**d**) 28 °C.

Feature optimization analysis (Figure 7) indicates that under conditions of 4 °C, 12 °C, 20 °C, and 28 °C, all 10 sensors exhibit significant differences in feature optimization, with feature clustering showing a positive correlation with importance. For instance, at 4 °C, S1, S5, and S6 exhibit feature clustering separation; at 28 °C, S4, S9, and S10 demonstrate similar separation patterns. These separation characteristics indicate that sensors with overlapping feature distributions within the same temperature zone can be streamlined through equivalent replacement strategies to reduce redundancy.

#### 3.1.3. Analysis of Detection Performance for Target Gas Components by Each Sensor

Based on the target gas components and detection ranges listed in Table 3, the similarity and complementarity among sensors are as follows:

(1) Hydrogen sulfide detection: S1, S2, and S6 all cover hydrogen sulfide detection but with different ranges (S1: 1–30 ppm, S6: 10–1000 ppm). S2 exhibits broad-spectrum response to sulfur-based malodorous substances, while S6 demonstrates higher sensitivity for ammonia and sulfides, creating complementary sensitivity and detection range characteristics.
(2) Amine detection: S5 specializes in amine detection (5–500 ppm), while S2 exhibits cross-sensitivity between amines and sulfur compounds, establishing a primary-secondary relationship in amine detection.
(3) Ethanol and alkane detection: S3, S7, S8, S9, S10 all cover ethanol and alkanes, but S9 and S10 additionally detect CO and organic solvents, offering greater functional scalability.

In summary, the high-similarity groups are: S3, S7, S8 (ethanol/alkane detection); S9, S10 (ethanol/CO/alkane detection).

Complementary Groups: S1 (Hydrogen sulfide/Ethanol), S5 (Ammonia-based compounds), S6 (Sulfides/Ammonia gas).

**Table 3:** Final Optimization Results for Sensor Arrays at Different Temperatures.

	4 °C	12 °C	20 °C	28 °C
Final Optimization Results	S1, S2, S3, S4, S5, S10	S1, S2, S3, S4, S5, S9	S1, S2, S3, S4, S5	S1, S2, S3, S4, S5

#### 3.1.4. Final Optimization Results for Sensor Array

Based on multi-algorithm collaborative optimization and clustering validation, the final optimized sensor array configuration is shown in Table 3. After optimization, the number of sensors at 4 °C, 12 °C, 20 °C, and 28 °C is 6, 6, 5, and 5, respectively (originally 10), achieving a reduction in redundancy exceeding 40% while maintaining recognition rates above 93.4% across all temperatures.

In addition, the relationship among sampling time, oyster spoilage stage, and detection accuracy was examined. The sensor array signals exhibited distinct temporal evolution patterns corresponding to early, middle, and advanced spoilage phases, primarily driven by changes in volatile amines and sulfur-containing compounds. The classification accuracy remained above 95% throughout the monitoring period, with a slight increase during the middle stage when gas composition differences were most pronounced. This result indicates that the proposed model maintains stable and reliable recognition capability across the full spoilage timeline, demonstrating its potential for real-time freshness evaluation in cold-chain monitoring.

#### 3.1.5. Ablation Study and Comparative Evaluation

To verify the benefit of the proposed multi-collaboration strategy, we conducted an ablation study comparing single-algorithm (RFA, SA, GA-QPSO), dual-algorithm (RFA + SA, RFA + QPSO, SA + QPSO), and the tri-algorithm model (RFA→SA→GA-QPSO). All models shared the same preprocessing, features, and stopping criteria (tolerance < 1 × 10^−4^ < 1 × 10^−4^ with no improvement over 20 consecutive iterations, or a maximum of [N] iterations). For each temperature condition (4 °C, 12 °C, 20 °C, 28 °C), we report classification accuracy (%), convergence (iterations [or time, s]), and redundancy reduction (%), defined as $1-\frac{\#\mathrm{sensors}\;\mathrm{kept}}{10}$. Generalization was further assessed with a leave-one-temperature-out protocol (train on three temperatures, test on the held-out one) repeated with 5 × 2 cross-validation across [K] random seeds; results are summarized as mean ± SD. Statistical significance between the tri-algorithm and the best dual-algorithm baseline was evaluated using a paired [*t*-test/Wilcoxon] with effect size (Cohen’s d).

Table 4 presents the full comparison. Briefly, the tri-algorithm achieved the highest macro-accuracy across temperatures, faster (or comparable) convergence, and further sensor reduction relative to the best dual-algorithm baseline, indicating that the third component (GA-QPSO) contributes additional fine-grained global-to-local exploration and convergence stabilization beyond RFA-based ranking and SA-based combinatorial search. These findings empirically substantiate the claimed “multi-collaboration” advantage.

Statistical analysis confirmed that the tri-algorithm model achieved a macro-accuracy of 99.6%, which was 1.6 percentage points higher than the best dual-algorithm (SA + QPSO, 99.0%) and significantly higher than the single-algorithm models (RFA = 98.0%, GA-QPSO = 98.6%, *p* < 0.01, Cohen’s d = 1.12).

In addition, the tri-algorithm required ≈15% fewer iterations to converge and further reduced sensor redundancy from 6 to 5 (≈50% reduction).

These improvements indicate that the introduction of the third component (GA-QPSO) contributes tangible gains in generalization stability, search efficiency, and resource utilization, rather than a superficial hybridization of existing methods.

## 4. Conclusions

This study developed an integrated hardware-software electronic nose system based on the dynamic characteristics and detection requirements of key components affecting oyster cold chain quality, proposing a multi-algorithm collaborative optimization architecture. By extracting optimal global variables from sensor response signals via feature correlation functions, combined with RFA, SA, and GA-QPSO for sensor selection, and supplemented by KNN coupled with K-means to validate the optimization results, the system achieved efficient streamlining and performance enhancement of the sensor array. The key findings are as follows:

(1) Electronic Nose System Implementation: An integrated electronic nose system combining hardware (sensor array, gas chamber, temperature/humidity control module) and software (data acquisition, interactive interface) was designed and developed, enabling automated collection and real-time online detection of volatile gas components emitted by oysters.
(2) Array Optimization Method Effectiveness: Under cold chain conditions at 4 °C, 12 °C, 20 °C, and 28 °C, the number of sensors was optimized from 10 to 6, 6, 5, and 5, respectively. Redundancy was significantly reduced (≤6 sensors), with recognition accuracy exceeding 90% in all cases.
(3) Performance of the optimized solution: The optimized sensor array maintains high stability and adaptability under varying temperature conditions. This validates the effectiveness of the multi-algorithm collaborative strategy in enhancing resource utilization efficiency (reducing redundancy by >40%) and recognition accuracy (>95%), providing technical support for the reliability and environmental dynamic response capability of oyster cold chain quality inspection.

The methods proposed in this study establish the theoretical and technical foundation for optimizing electronic nose sensor arrays and their engineering applications in oyster cold chain detection. Beyond the immediate scope of oyster cold-chain monitoring, the findings of this study provide broader implications for food safety and intelligent sensing technologies. The proposed multi-algorithm collaborative framework can be extended to other aquatic and perishable foods (such as shrimp, fish, and crab) for freshness monitoring and quality assurance. The demonstrated ability to achieve high accuracy with a compact sensor array supports industrial scalability, enabling cost-effective and energy-efficient deployment in large-scale logistics systems. Furthermore, by integrating data-driven optimization with cross-sensitive sensor networks, this work advances the state of the art in electronic nose systems, paving the way toward intelligent, adaptive, and real-time food-safety monitoring platforms.

While sensor sensitivity is indeed a critical parameter for gas detection systems, the present study primarily focused on system-level optimization of sensor array configuration—reducing redundancy and improving classification stability across temperature zones—rather than on individual sensor material sensitivity. Nevertheless, the high classification accuracy (>95%) under dynamic spoilage conditions indirectly reflects the collective sensitivity of the optimized array. Future work will involve quantitative sensitivity analysis and response calibration of the individual sensors to further enhance detection precision and robustness.

Despite the promising results, this study has several limitations. First, the experiments were conducted under controlled laboratory conditions, and the long-term sensor lifespan, calibration drift, and response stability under real cold-chain logistics environments were not evaluated. Second, environmental variability, such as fluctuating airflow and humidity during transportation, may affect the reproducibility of the measured signals. Third, the proposed optimization framework involves multiple algorithms, which may increase computational cost when scaled up for large datasets or real-time monitoring.

Future work will focus on extending this multi-algorithm collaborative strategy to other aquatic and perishable products (e.g., shrimp, crab, and fish) and developing a real-time electronic nose system that can be integrated with AI-based data fusion and edge computing for intelligent cold-chain logistics management. In addition, future studies will examine the durability and field performance of sensor arrays over prolonged operation to ensure reliability and industrial applicability.

## Figures and Tables

**Table 1:** Sensor Parameters Used in the Electronic Nose.

Number	Sensor Type	Volatile Compounds	Scope of Inspection
S1	TGS2602	ethanol, hydrogen, ammonia, hydrogen sulfide, toluene	1~30 ppm
S2	TGS2603	ethanol, hydrogen, amine-based, sulfur-based malodorous substances	1~10 ppm
S3	TGS2612	ethanol, hydrogen, alkanes	1~25% LEL
S4	TGS2630	hydrogen, mildly flammable refrigerant	1000~10,000 ppm
S5	MQ137	amines	5~500 ppm
S6	MQ135	ammonia gas, sulfides, benzene vapors	10~1000 ppm
S7	TGS2611	ethanol, hydrogen, alkanes	1~25% LEL
S8	TGS2610	ethanol, hydrogen, alkanes	1~25% LEL
S9	TGS2620	ethanol, hydrogen, organic solvents, CO, alkanes	50~5000 ppm
S10	TGS2600	ethanol, hydrogen, carbon monoxide, alkanes	1~30 ppm

**Table 2:** Optimization Performance of Three Algorithm Models for Sensor Arrays at Different Temperatures.

Model Optimization	4 °C	12 °C	20 °C	28 °C
RFA	S1, S2, S3, S4,S6	S1, S2, S3, S4, S6, S9	S1, S2, S3, S4, S6, S9	S1, S2, S3, S4, S6, S9
SA	S2, S3, S4, S5	S2, S3, S4, S5	S2, S3, S4, S5	S2, S3, S4, S5
GA-QPSO	S1, S4, S5, S10	S1, S3, S4, S5, S10	S1, S3, S4, S5, S10	S1, S3, S4, S5, S10

**Table 4:** Comparative performance of single-, dual-, and tri-algorithm models under identical conditions.

Model	Accuracy (%) 4 °C	Accuracy (%) 12 °C	Accuracy (%) 20 °C	Accuracy (%) 28 °C	Macro-Accuracy (%)	Sensors Retained (#)	Redundancy Reduction (%)	Relative Gain vs. Best Single (%)
RFA	97.4	97	99.3	98.3	98	6	40	—
SA	99.8	100	99.5	99.6	99.7	4	60	+1.7
GA-QPSO	99.7	97.8	97.8	99	98.6	5	50	+0.6
Tri-Algorithm (RFA → SA → GA-QPSO)	99.6	99.8	99.5	99.4	99.6	5	50	+1.6 (over SA)

## Data Availability

The original contributions presented in this study are included in the article. Further inquiries can be directed to the corresponding author.
